# Augmented Reality-Assisted Placement of Ommaya Reservoir for Cyst Aspiration: A Case Report

**DOI:** 10.7759/cureus.52383

**Published:** 2024-01-16

**Authors:** Joshua Olexa, Annie Trang, Kevin Kim, Maureen Rakovec, Jordan Saadon, Whitney Parker

**Affiliations:** 1 Neurosurgery, University of Maryland School of Medicine, Baltimore, USA

**Keywords:** mixed reality, cyst, image guidance, ommaya reservoir, augmented reality

## Abstract

Image guidance technologies can significantly improve the accuracy and safety of intracranial catheter insertions. Augmented reality (AR) allows surgeons to visualize 3D information overlaid onto a patient's head. As such, AR has emerged as a novel image guidance technology that offers unique advantages when navigating intracranial targets.

A 71-year-old woman with a history of brain metastasis from breast cancer and prior resection surgery and chemotherapy presented with altered mental status and generalized weakness worse on her left side. Magnetic resonance imaging (MRI) demonstrated right frontotemporoparietal edema with a contrast-enhancing mass. MR perfusion confirmed an active tumor with an enlarging right temporal pole cyst. A cyst aspiration was performed via Ommaya reservoir placement. Neuro-navigation (BrainLab, Munich, Germany) and AR navigation were used to plan the trajectory from the temporal gyrus to the cyst. Post-operative computed tomography (CT) demonstrated good placement of the reservoir, reconstitution of the temporal horn of the lateral ventricle with decreased external mass effect, and no areas of hemorrhage.

AR has tremendous potential in the field of neurosurgery for improving the accuracy and safety of procedures. This case demonstrates an encouraging application of AR and can serve as an example to drive expanded clinical use of this technology.

## Introduction

Advancements in medical technology continue to reshape the landscape of neurosurgery, offering innovative solutions to complex challenges. Augmented reality (AR) has emerged as a promising tool, providing surgeons with real-time visualization and enhanced spatial awareness during intricate procedures. This technology allows for the precise localization of lesions and accurate trajectory planning. By superimposing patient-specific imaging data onto the surgical field, AR enables surgeons to navigate complex anatomical structures with enhanced accuracy and efficiency [1-3]. The case presented herein details a specific instance where AR was employed for the placement of an Ommaya reservoir for the aspiration of a temporal pole cyst. The integration of AR technology facilitated improved visualization of the cystic lesion's location, optimized surgical planning, and trajectory guidance for needle placement during aspiration. 

## Case presentation

Segmenting anatomical structures

A 3D model of the patient’s anatomy was generated from the patient’s preoperative MRI scan. Anatomy of interest, that is, the whole brain, tumor, cyst, ventricles, and vasculature, were manually segmented using ITK-SNAP (Penn Image Computing and Science Laboratory {PICSL}, University of Pennsylvania, Philadelphia, Pennsylvania). All segmented structures were merged and assembled into a 3D digital model.

Augmented reality technology platform

The AR software application used for the case was developed by Hoth Intelligence (Philadelphia, Pennsylvania) and functions on the Microsoft Hololens 2 HMD (Redmond, Washington). The Microsoft Hololens 2 is an optical see-through head-mounted display that superimposes virtual content (i.e., holograms, images, screens) onto the surgeons’ surgical field of view [4-6].

3D model registration to patient

The AR system uses a markerless (fiducial-less), rapid registration process. The headset contains various sensors that capture information from the patient’s head and face. Information (i.e., head shape, key points of face) captured from the headset sensors (depth sensor, RBG camera, and stereo sensor) is then aligned to the corresponding points on the 3D model that was generated from the preoperative MRI scan. This alignment results in the registration and overlay of the patients’ 3D anatomical model onto the patients’ head.

Mixed reality viewing and surgical planning

**Figure 1:**
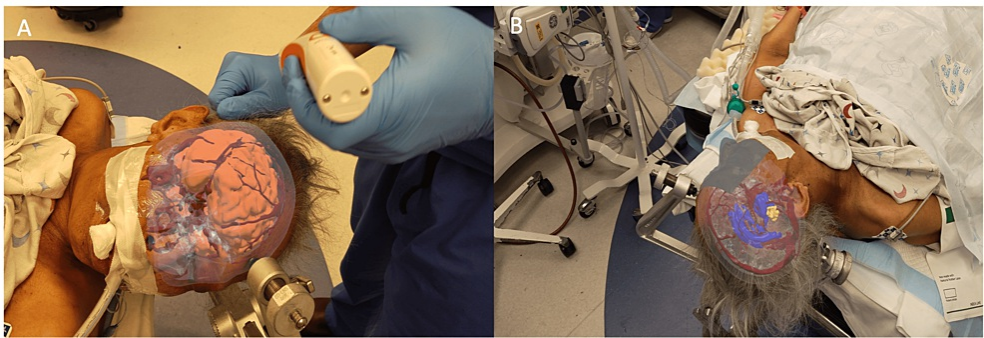
User’s view through the augmented reality headset displaying a 3D model overlaid onto the patient's head from different perspectives. (A) 3D model showing brain (orange) and vasculature (red). (B) 3D model with a transparent view showing vasculature (red), tumor (yellow), ventricles (blue), and cyst (blue).

Once the 3D model is registered to the patient, the surgeon visualizes the digital 3D model overlaid onto the patient’s head. The 3D model remains fixed on the head, allowing the physician to freely move around the patient to visualize the 3D anatomy from different perspectives (Figure 1). Additionally, AR technology provides the surgeon with planning tools. In this case, the surgeon placed virtual trajectories towards the intracranial cyst to assist with placing the Ommaya catheter.

Ethical approval

Institutional Review Board (IRB) approval for the application of this technology was obtained via the University of Maryland’s IRB committee (IRB number HP-00104849) and allowed for intraoperative use for presurgical planning in the setting of this technology. Confirmation was obtained with appropriate consent.

Case presentation

**Figure 2:**
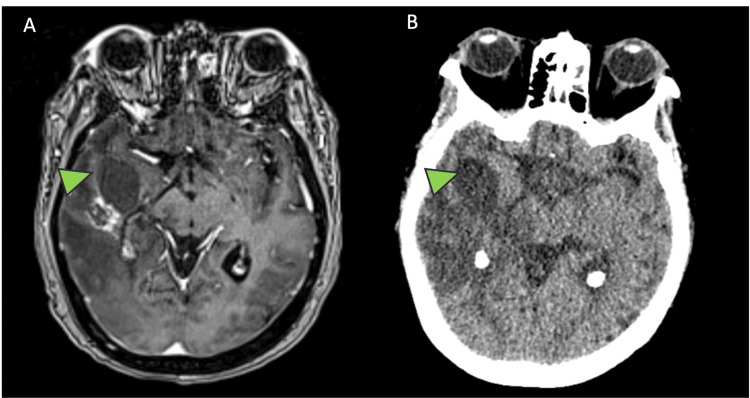
Pre-operative patient imaging. (A) Preoperative T1 MRI. (B) Preoperative CT scan showing the presence of cyst and contrast-enhancing lesion. The green arrow indicates the location of the temporal pole cyst.

A 71-year-old woman with a history of brain metastasis from breast cancer and prior resection surgery and chemotherapy presented with altered mental status and generalized weakness worse on her left side. MRI and CT imaging demonstrated right frontotemporoparietal edema with a contrast-enhancing mass and an enlarging right temporal pole cyst (Figure 2). Given the high risk with further radiation and the patient’s coagulopathy and thrombocytopenia, a cyst aspiration was performed.

Augmented reality surgical planning

**Figure 3:**
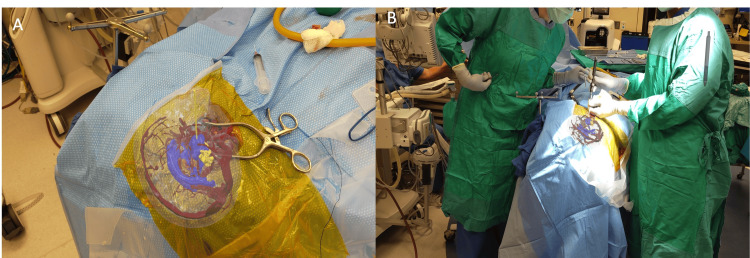
User’s view through the augmented reality (AR) headset displaying a 3D model overlaid onto the patient's head intraoperatively. (A) While wearing the headset, the surgeon placed a trajectory  (yellow line) towards the cyst. (B) The AR system was used in conjunction with traditional neuronavigation.  The navigation probe was aligned with an AR-placed trajectory line to guide catheter placement.

After the AR system was used to register 3D reconstructions of the patient's anatomy onto the patient's head the surgical team visualized the location of the cyst and planned insertion trajectories. The AR display included a 3D reconstruction of the brain, vasculature, ventricular, tumor, and cyst. Following registration, an AR trajectory planning tool was used. The planning tool is a virtual pointer that is used to place trajectories in the surgeon’s real-world field of view. For the case, the surgeon used the AR system to place and plan a trajectory towards the cyst which was then confirmed and followed in conjunction with traditional neuronavigation to guide catheter placement towards the cyst (Figure 3).

Operative and post-operative course

**Figure 4:**
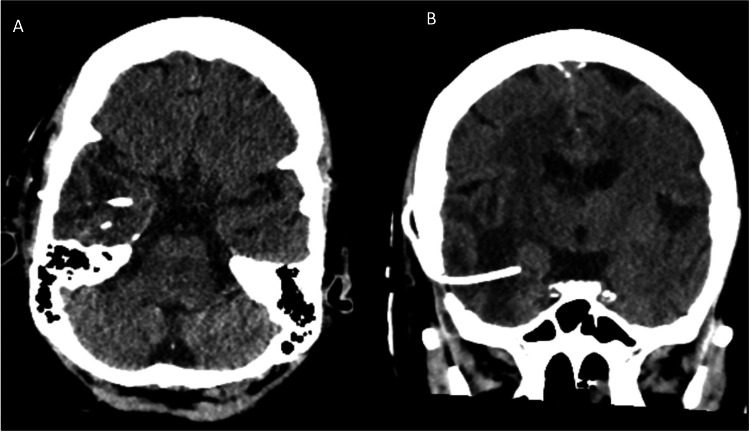
Post-operative imaging. Axial (A) and coronal (B) view of post-operative CT demonstrating placement of Ommaya reservoir.

Augmented reality navigation and neuro-navigation (BrainLab, Munich, Germany) were used to plan the trajectory from the temporal gyrus to the cyst. The augmented reality view allowed for 3D visualization of the cyst as well as a preplanned trajectory line towards a target location within the cyst. After drilling a bur hole, a neuro-navigation stylet was used to follow the trajectory displayed by the augmented reality system. The Ommaya catheter was inserted at a depth of approximately 4cm without breaching the lateral ventricle. Post-operatively, head CT demonstrated good placement of the reservoir, reconstitution of the temporal horn of the lateral ventricle with decreased external mass effect, and no areas of hemorrhage (Figure 4).

## Discussion

AR is rapidly emerging as a promising technology in the field of neurosurgery. As such, it is imperative to continue to demonstrate and document the preclinical and clinical use of the technology in order to drive further adoption. Here we describe the implementation of a novel AR technology during the placement of an Ommaya catheter for cyst aspiration. This marks a significant advancement in the use of mixed reality technology during neurosurgical procedures. By integrating patient-specific imaging data, AR allows surgeons to access three-dimensional representations of the patient's anatomy, enabling them to strategize the catheter's trajectory and anticipate potential obstacles or variations in anatomical structures.

The use of traditional AR systems is largely focused on presurgical planning in which a user can visualize and interact with 3D anatomic models prior to beginning the case. These systems are helpful for understanding spatial relationships of complex anatomy [7,8]. However, the ability to overlay said 3D models onto the patient's head represents a unique advantage. Reports have described the use of AR to register 3D models onto a patient's head however, there are several technical characteristics of the system used for this case that are worth highlighting [9-13]. First, the system is markerless and thus can register preoperative imaging data onto the patient without the need for fiducials. Second, the registration process takes approximately ten seconds. Finally, the entire system operates solely out of the AR headset without the need for external cameras and computer screens. As such the small footprint and rapid workflow make it an ideal surgical planning tool.

In this case, the system was used in conjunction with traditional navigation. With this added visualization, the surgeon was able to more easily orient themselves with the patient’s anatomy as opposed to extrapolating 3D locations from the traditional 2D navigation views. Trajectory planning with traditional navigation systems can also be challenging. While the trajectory was confirmed using traditional navigation, the ability to overlay a virtual trajectory directly onto the patient's head allowed the surgeon to more easily and more rapidly plan the insertion path. Altogether the system provided a unique visualization of the anatomy to ensure proper patient positioning, localization of anatomy, and trajectory planning.

While the benefits of the system have been described, there are several limitations worth noting in this report. A measurable benefit of the AR system is not obtainable from this work as the results of this case were not compared with those from a similar case in which AR was not used. Additionally, while AR may have advantages over traditional neuronavigation, a direct comparison between the two systems cannot be made from this report. Conducting robust preclinical and clinical studies to evaluate the efficacy and safety of AR-guided procedures compared to conventional methods is crucial. Accuracy metrics, outcome data, and comparative analyses will be instrumental in establishing the superiority and clinical benefits of AR technology in this context.

## Conclusions

This case report intends to shed light on the feasibility, advantages, and potential challenges associated with employing AR in neurosurgical interventions. We aim to contribute to the evolving field of neurosurgery and emphasize the potential of AR as a valuable adjunct for enhancing surgical precision and patient care. While challenges exist, ongoing technological advancements and comprehensive validation studies may further establish its role as an invaluable tool in neurosurgical practice.
